# Bis(2-amino-4-methyl-6-oxo-3,6-di­hydro­pyrimidin-1-ium) sulfate monohydrate

**DOI:** 10.1107/S1600536814012513

**Published:** 2014-06-04

**Authors:** Sarra Soudani, Emmanuel Wenger, Christian Jelsch, Cherif Ben Nasr

**Affiliations:** a Laboratoire de Chimie des Matériaux, Faculté des Sciences de Bizerte, 7021 Zarzouna, Tunisia; b Cristallographie, Résonance Magnétique et Modélisations (CRM2), UMR CNRS 7036, Institut Jean Barriol, Université de Lorraine, BP 70239, Bd des Aiguillettes, 54506 Vandoeuvre-les-Nancy, France; c Laboratoire de Chimie des Matériaux, Faculté des sciences de Bizerte, 7021 Zarzouna, Tunisia

## Abstract

In the title hydrated mol­ecular salt, 2C_5_H_8_N_3_O^+^·SO_4_^2−^·H_2_O, the components are linked by N—H⋯O_s_ and O_w_—H⋯O_s_ (s = sulphate, w = water) hydrogen bonds, generating a layer by *a*+*b*+*c* and 2*a*−*b* translations. The cations are arranged nearly in parallel and show displaced π–π stacking centroid–centroid distance = 4.661 (2) Å between adjacent layers.

## Related literature   

For the applications of oxoanion compounds, see: Vollano *et al.* (1984 ▶); Molloy (1988 ▶). For graph-set motifs, see: Bernstein *et al.* (1995 ▶). For the stability of the quinonic and phenolic form in polar solvents, see: Fragoso *et al.* (2010 ▶). For C—N single bond lengths, see: Yang *et al.* (1995 ▶); Grobelny *et al.* (1995 ▶). For the geometrical characteristics of the sulfate anion, see: Das *et al.* (2009 ▶); Norquist *et al.* (2005 ▶). 
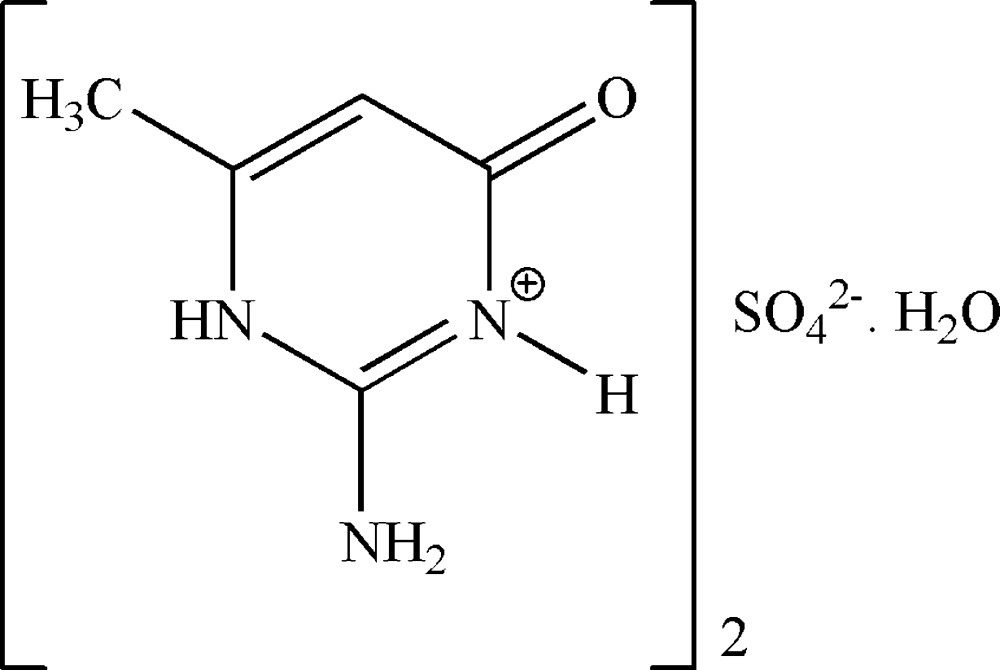


## Experimental   

### 

#### Crystal data  

2C_5_H_8_N_3_O^+^·SO_4_^2−^·H_2_O
*M* *_r_* = 366.36
Triclinic, 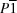

*a* = 6.797 (5) Å
*b* = 10.339 (6) Å
*c* = 12.110 (7) Å
α = 113.480 (5)°
β = 91.009 (7)°
γ = 98.906 (5)°
*V* = 768.2 (8) Å^3^
*Z* = 2
Mo *K*α radiation
μ = 0.26 mm^−1^
*T* = 100 K
0.27 × 0.19 × 0.12 mm

#### Data collection  

Bruker APEXII CCD diffractometer
Absorption correction: multi-scan (*SADABS*; Sheldrick, 2008 ▶) *T*_min_ = 0.92, *T*_max_ = 0.97
5784 measured reflections
5784 independent reflections
5347 reflections with *I* > 2σ(*I*)

#### Refinement  

*R*[*F* ^2^ > 2σ(*F* ^2^)] = 0.081
*wR*(*F* ^2^) = 0.131
*S* = 1.35
5784 reflections
289 parameters
15 restraints
H atoms treated by a mixture of independent and constrained refinement
Δρ_max_ = 0.64 e Å^−3^
Δρ_min_ = −0.38 e Å^−3^

### 

Data collection: *APEX2* (Bruker, 2007 ▶); cell refinement: *SAINT* (Bruker, 2012 ▶); data reduction: *SAINT*; program(s) used to solve structure: *SHELXS97* (Sheldrick, 2008 ▶); program(s) used to refine structure: *SHELXL97* (Sheldrick, 2008 ▶); molecular graphics: *ORTEP-3 for Windows* (Farrugia, 2012 ▶); software used to prepare material for publication: *SHELXL97*.

## Supplementary Material

Crystal structure: contains datablock(s) global, I. DOI: 10.1107/S1600536814012513/bg2531sup1.cif

Structure factors: contains datablock(s) I. DOI: 10.1107/S1600536814012513/bg2531Isup2.hkl

Supporting information file. DOI: 10.1107/S1600536814012513/bg2531Isup3.cml

CCDC reference: 1005732

Additional supporting information:  crystallographic information; 3D view; checkCIF report

## Figures and Tables

**Table 1:** Hydrogen-bond geometry (Å, °)

*D*—H⋯*A*	*D*—H	H⋯*A*	*D*⋯*A*	*D*—H⋯*A*
N1—H1*A*⋯O6^i^	0.87 (3)	2.02 (3)	2.856 (3)	161 (3)
N1—H1*B*⋯O4^ii^	0.83 (3)	2.01 (3)	2.847 (4)	178 (2)
N2—H2⋯O6^iii^	0.91 (3)	1.88 (2)	2.749 (4)	161 (2)
N7—H7⋯O7^ii^	0.83 (3)	1.91 (3)	2.730 (4)	170 (3)
N11—H11*A*⋯O5^iii^	0.87 (3)	1.91 (3)	2.782 (6)	178 (3)
N11—H11*B*⋯O5^iv^	0.89 (3)	2.07 (3)	2.767 (3)	134 (2)
N12—H12⋯O8^iv^	0.92 (3)	1.87 (3)	2.771 (4)	170 (3)
N17—H17⋯O4^iii^	0.82 (3)	1.92 (3)	2.746 (6)	177 (3)
O8—H8*A*⋯O13^v^	0.86 (3)	1.94 (3)	2.750 (5)	158 (3)
O8—H8*B*⋯O7	0.83 (3)	2.02 (3)	2.839 (4)	173 (3)
C4—H4⋯O3^vi^	0.97 (2)	2.54 (2)	3.485 (3)	165 (2)
C6—H6*C*⋯O13^ii^	0.97	2.51	3.440 (3)	162 (2)
C16—H16*A*⋯O3^vii^	0.95	2.58	3.499 (5)	163 (2)

Symmetry codes: (i) 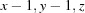; (ii) 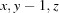; (iii) 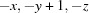; (iv) 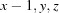; (v) 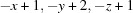; (vi) 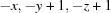; (vii) 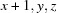.

**Fig. 1.**
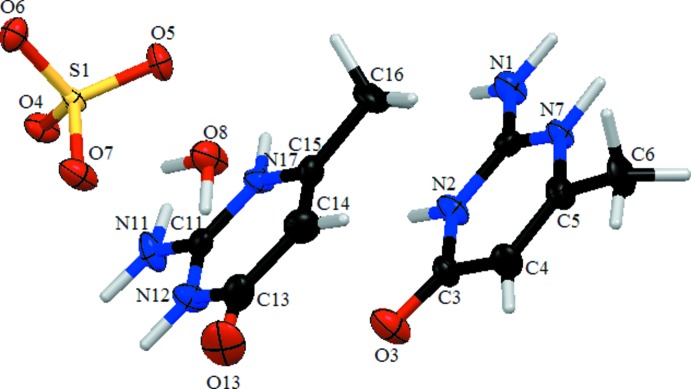
ORTEP view of the title compound, showing 50% probability thermal displacement ellipsoids and spheres for the H atoms.

**Fig. 2.**
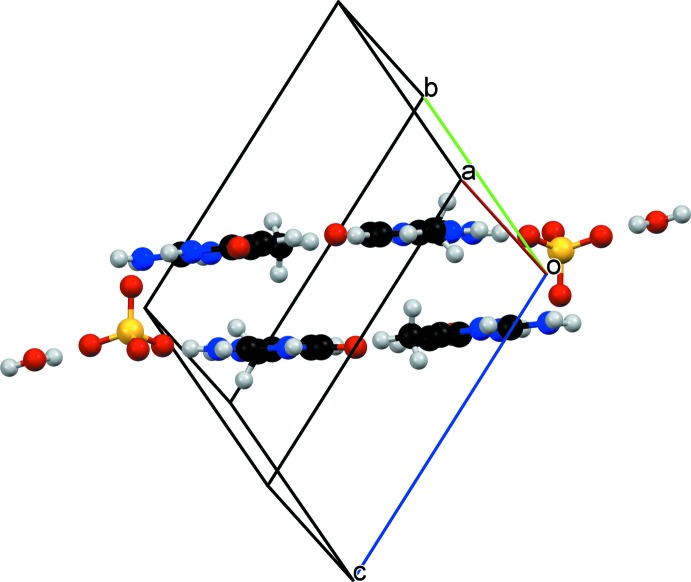
View showing the planes formed by the two independant cations molecules. The sulfate anions form the strongest interactions between two parallel layers.

**Fig. 3.**
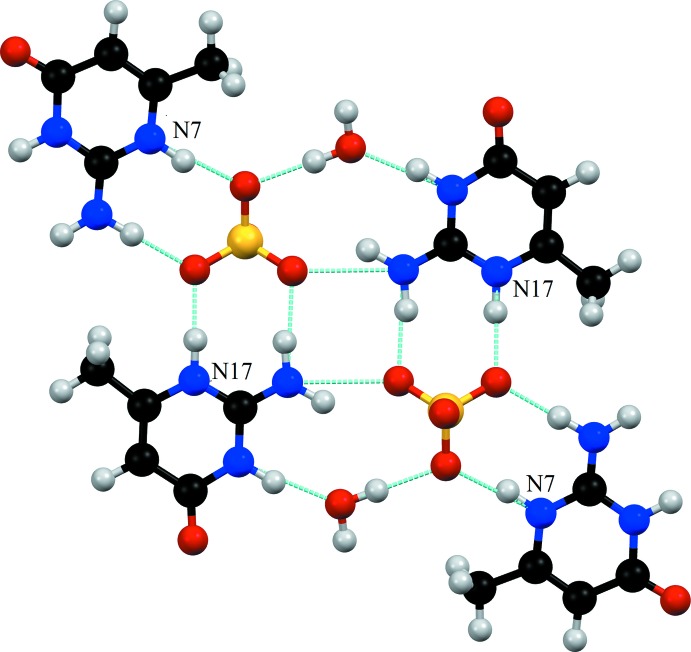
View perpendicular to Fig. 2 showing hydrogen bonds stabilizing the structure of the title material in the layer containing the cations.

**Fig. 4.**
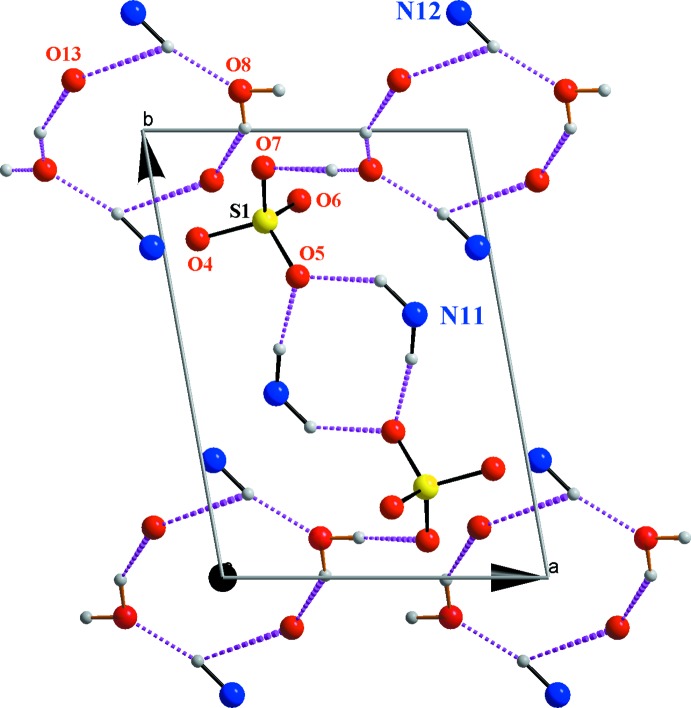
View along the c-axis showing O—H···O and N—H···O hydrogen bonds forming two kinds of 8-membered rings.

## supplementary crystallographic information

### S1. Comment

Chemists and physicists of the solid state have shown an increasing interest in the study of oxoanion compounds containing organic cations in recent years owing to their applications in various fields (Vollano *et al.*, 1984; Molloy, 1988). Here, we report the synthesis and the crystal structure of the title compound 2(C_5_H_8_N_3_O), O_4_S, H_2_O. The asymmetric unit of this salt contains two molecules of 2-amino-6-methylpyrimidin-4-(1*H*)-one, one sulfate anion and one water molecule (Fig. 1). The two independent aromatic cycles of the asymmetric unit are nearly parallel as they form an angle of 5.8°. The shortest distance between non-H atoms of two cations in parallel displaced π- staking is *d*(O3···N17) = 3.375 (2) Å. When the two cation molecules are viewed along the two centroids, the 6 atom positions of the two cycles appear superposed after a rotation of 60° (Fig. 1). The crystal structure of the title material consists of a network of the different constituents connected by a set of hydrogen bonds (Table 1). Furthermore, in the crystal packing, the two cations are arranged in layers (Fig. 2). The sulfate anion forms the strongest interactions between two parallel layers, as each sulfate moiety interacts with the five cations *via* seven N—H···O hydrogen bonds. The hydrogen bond network in the cations layer is shown in Fig. 3. The water molecule is acceptor in one N—H···O hydrogen bond and donor in two O—H···O interactions with the sulfate anion and carbonyl group. In the atomic arrangement of the title compound, the sulfate anions are located close to the *z*=0 plane. Along the *a* diretion, they are interconnected *via* a same NH_2_ group. In the crystal structure, various graph set motifs (Bernstein *et al.*, 1995) are apparent including *R*_2_^4^ (8) and *R*_3_^6^ (8) loops (Fig. 4). An examination of Table 2 data shows that the distance values of C3—O3 (1.229 (2) Å) and C13—O13 (1.226 (2) Å) can be attributed as having clear double bond character indicating that the title compound is present as the keto tautomer of the 2-amino-6-methyl-4-pyrimidinol(sheme. 2). This observation agrees with the literature data which show that in polar solvents, the quinonic form is more stable than the phenolic one (Fragoso *et al.*, 2010). The C—N bond distances of the NH_2_ groups are C1—N1 (1.315 (2) Å), and C11—N11 (1.318 (2) Å) which are short for C—N single bonds, but still not quite as contracted as one would expect for a fully established C=N double bond. These bond length features are consistent with an imino resonance form as it is commonly found for a C—N single bond involving *sp*^2^ hybridized C and N atoms, (Yang *et al.*, 1995; Grobelny *et al.*, 1995). The S—O bond lengths and the O—S—O bond angles in the sulfate anion are not perfectly equivalent (Das *et al.*, 2009; Norquist *et al.*, 2005), but vary with the environment around the O atoms. In the title compound, the S—O distances are spread between 1.4759 (14) and 1.4949 (16) Å. The O—S—O bond angles range from 108,97 (8) to 110.24 (9)°. All these geometrical parameters indicate relatively little distortion from a regular tetrahedron.

### S2. Experimental

Commercial 2-amino-4-hydroxy-6-methylpyrimidine (50 mg, 0.4 mmol) dissolved in ethanol (10 ml) was slowly added under stirring to 0.05 mol of sulfuric acid in 20 ml of water. The obtained solution was left to stand at room temperature. The slow evaporation of solvent leads to the formation of colourless single crystals of (I), stable in air and suitable for X-ray diffraction analysis.

### S3. Refinement

The structure was refined with *SHELXL97* (Sheldrick, 2008). The coordinates of the H atoms were refined with *U*_iso_(H) = 1.2Ueq(*X*) except for the methyl groups for which *U*_iso_(H) was set to 1.5Ueq(*X*), where *X* is the parent atom. The C—H distances in the two methyl groups were restraint to be similar (sigma=0.01).

### Crystal data

**Table:**

2C_5_H_8_N_3_O^+^·SO_4_^2^^−^·H_2_O	*V* = 768.2 (8) Å^3^
*M**_r_* = 366.36	*Z* = 2
Triclinic, *P*1	*F*(000) = 384
Hall symbol: -P 1	*D*_x_ = 1.584 Mg m^−^^3^
*a* = 6.797 (5) Å	Mo *K*α radiation, λ = 0.71073 Å
*b* = 10.339 (6) Å	θ = 2.2–33.4°
*c* = 12.110 (7) Å	µ = 0.26 mm^−^^1^
α = 113.480 (5)°	*T* = 100 K
β = 91.009 (7)°	Prism, colourless
γ = 98.906 (5)°	0.27 × 0.19 × 0.12 mm

### Data collection

**Table:**

Bruker APEXII CCD diffractometer	5784 independent reflections
Radiation source: fine-focus sealed tube	5347 reflections with *I* > 2σ(*I*)
Mirror monochromator	*R*_int_ = 0.000
ω scans	θ_max_ = 33.1°, θ_min_ = 3.1°
Absorption correction: multi-scan (*SADABS*; Sheldrick, 2008)	*h* = 0→10
*T*_min_ = 0.92, *T*_max_ = 0.97	*k* = −15→15
5784 measured reflections	*l* = −18→18

### Refinement

**Table:**

Refinement on *F*^2^	Primary atom site location: structure-invariant direct methods
Least-squares matrix: full	Secondary atom site location: difference Fourier map
*R*[*F*^2^ > 2σ(*F*^2^)] = 0.081	Hydrogen site location: inferred from neighbouring sites
*wR*(*F*^2^) = 0.131	H atoms treated by a mixture of independent and constrained refinement
*S* = 1.35	*w* = 1/[σ^2^(*F*_o_^2^) + (0.044*P*)^2^ + 0.4148*P*] where *P* = (*F*_o_^2^ + 2*F*_c_^2^)/3
5784 reflections	(Δ/σ)_max_ < 0.001
289 parameters	Δρ_max_ = 0.64 e Å^−^^3^
15 restraints	Δρ_min_ = −0.38 e Å^−^^3^

### Special details

**Table:**

Geometry. All e.s.d.'s (except the e.s.d. in the dihedral angle between two l.s. planes) are estimated using the full covariance matrix. The cell e.s.d.'s are taken into account individually in the estimation of e.s.d.'s in distances, angles and torsion angles; correlations between e.s.d.'s in cell parameters are only used when they are defined by crystal symmetry. An approximate (isotropic) treatment of cell e.s.d.'s is used for estimating e.s.d.'s involving l.s. planes.
Refinement. Refinement of *F*^2^ against ALL reflections. The weighted *R*-factor *wR* and goodness of fit *S* are based on *F*^2^, conventional *R*-factors *R* are based on *F*, with *F* set to zero for negative *F*^2^. The threshold expression of *F*^2^ > σ(*F*^2^) is used only for calculating *R*-factors(gt) *etc*. and is not relevant to the choice of reflections for refinement. *R*-factors based on *F*^2^ are statistically about twice as large as those based on *F*, and *R*- factors based on ALL data will be even larger.

### Fractional atomic coordinates and isotropic or equivalent isotropic displacement parameters (Å^2^)

**Table:**

	*x*	*y*	*z*	*U*_iso_*/*U*_eq_	
S1	0.32594 (6)	0.79761 (4)	0.06036 (4)	0.00888 (9)	
O4	0.10950 (17)	0.75634 (13)	0.01545 (11)	0.0129 (2)	
O5	0.39545 (18)	0.67318 (13)	0.06868 (11)	0.0136 (2)	
O6	0.44256 (18)	0.84309 (13)	−0.02448 (11)	0.0137 (2)	
O7	0.34949 (19)	0.91648 (13)	0.18190 (11)	0.0161 (3)	
C1	−0.1015 (2)	0.07187 (17)	0.18509 (14)	0.0097 (3)	
N1	−0.1580 (2)	−0.04398 (16)	0.08537 (13)	0.0125 (3)	
N2	−0.2110 (2)	0.17792 (15)	0.22132 (13)	0.0113 (3)	
C3	−0.1599 (2)	0.30560 (18)	0.32540 (15)	0.0121 (3)	
O3	−0.2627 (2)	0.39938 (14)	0.34694 (12)	0.0188 (3)	
C4	0.0171 (3)	0.31382 (18)	0.39715 (15)	0.0128 (3)	
C5	0.1275 (2)	0.20844 (17)	0.35890 (15)	0.0109 (3)	
C6	0.3178 (3)	0.2130 (2)	0.42459 (17)	0.0171 (3)	
N7	0.0687 (2)	0.08908 (15)	0.25224 (13)	0.0108 (3)	
C11	−0.0959 (2)	0.61151 (17)	0.19155 (15)	0.0113 (3)	
N11	−0.2643 (2)	0.58800 (17)	0.12550 (14)	0.0145 (3)	
N12	−0.0358 (2)	0.73732 (16)	0.28733 (13)	0.0138 (3)	
C13	0.1415 (3)	0.76947 (19)	0.36118 (16)	0.0151 (3)	
O13	0.1845 (2)	0.88853 (14)	0.44418 (12)	0.0226 (3)	
C14	0.2552 (3)	0.65558 (19)	0.33060 (16)	0.0153 (3)	
C15	0.1953 (2)	0.53064 (18)	0.23358 (15)	0.0119 (3)	
C16	0.3067 (3)	0.4085 (2)	0.18954 (17)	0.0149 (3)	
N17	0.0195 (2)	0.51065 (15)	0.16497 (13)	0.0111 (3)	
O8	0.6783 (2)	0.91155 (15)	0.32711 (12)	0.0190 (3)	
H1A	−0.274 (4)	−0.063 (3)	0.046 (2)	0.020 (6)*	
H1B	−0.082 (4)	−0.104 (3)	0.064 (2)	0.019 (6)*	
H2	−0.310 (4)	0.173 (3)	0.168 (2)	0.028 (7)*	
H4	0.060 (4)	0.398 (2)	0.472 (2)	0.019 (6)*	
H6A	0.344 (3)	0.302 (3)	0.4979 (18)	0.032 (7)*	
H6B	0.425 (2)	0.212 (3)	0.379 (2)	0.031 (7)*	
H6C	0.306 (4)	0.130 (3)	0.444 (2)	0.038 (8)*	
H7	0.143 (4)	0.028 (3)	0.230 (2)	0.029 (7)*	
H11A	−0.303 (4)	0.505 (3)	0.065 (3)	0.029 (7)*	
H11B	−0.337 (4)	0.657 (3)	0.143 (2)	0.026 (6)*	
H12	−0.118 (4)	0.804 (3)	0.305 (2)	0.027 (6)*	
H14	0.374 (4)	0.672 (3)	0.378 (2)	0.023 (6)*	
H16A	0.417 (3)	0.424 (3)	0.245 (2)	0.031 (7)*	
H16B	0.351 (3)	0.397 (2)	0.1108 (18)	0.024 (6)*	
H16C	0.220 (4)	0.3214 (14)	0.178 (2)	0.021 (6)*	
H17	−0.020 (4)	0.432 (3)	0.109 (3)	0.030 (7)*	
H8A	0.694 (5)	0.983 (3)	0.396 (3)	0.041 (8)*	
H8B	0.577 (4)	0.914 (3)	0.290 (3)	0.030 (7)*	

### Atomic displacement parameters (Å^2^)

**Table:**

	*U*^11^	*U*^22^	*U*^33^	*U*^12^	*U*^13^	*U*^23^
S1	0.00752 (16)	0.00803 (17)	0.00983 (17)	0.00195 (12)	−0.00157 (12)	0.00220 (13)
O4	0.0068 (5)	0.0115 (5)	0.0166 (6)	0.0011 (4)	−0.0022 (4)	0.0021 (4)
O5	0.0146 (6)	0.0110 (5)	0.0163 (6)	0.0039 (4)	−0.0024 (5)	0.0062 (5)
O6	0.0106 (5)	0.0167 (6)	0.0154 (6)	0.0004 (4)	−0.0017 (4)	0.0090 (5)
O7	0.0152 (6)	0.0146 (6)	0.0124 (6)	0.0066 (5)	−0.0038 (5)	−0.0022 (4)
C1	0.0081 (6)	0.0115 (7)	0.0100 (7)	0.0016 (5)	0.0006 (5)	0.0048 (6)
N1	0.0091 (6)	0.0125 (6)	0.0126 (6)	0.0027 (5)	−0.0017 (5)	0.0015 (5)
N2	0.0098 (6)	0.0124 (6)	0.0113 (6)	0.0043 (5)	−0.0009 (5)	0.0034 (5)
C3	0.0113 (7)	0.0123 (7)	0.0119 (7)	0.0020 (6)	0.0001 (6)	0.0041 (6)
O3	0.0181 (6)	0.0157 (6)	0.0202 (6)	0.0088 (5)	−0.0010 (5)	0.0028 (5)
C4	0.0133 (7)	0.0115 (7)	0.0116 (7)	0.0019 (6)	−0.0020 (6)	0.0026 (6)
C5	0.0099 (7)	0.0110 (7)	0.0111 (7)	0.0000 (5)	−0.0015 (5)	0.0044 (6)
C6	0.0141 (8)	0.0163 (8)	0.0184 (8)	0.0029 (6)	−0.0066 (6)	0.0048 (7)
N7	0.0088 (6)	0.0101 (6)	0.0117 (6)	0.0030 (5)	−0.0013 (5)	0.0022 (5)
C11	0.0107 (7)	0.0110 (7)	0.0121 (7)	0.0009 (6)	0.0004 (6)	0.0051 (6)
N11	0.0112 (6)	0.0115 (6)	0.0186 (7)	0.0032 (5)	−0.0040 (5)	0.0037 (6)
N12	0.0143 (7)	0.0109 (6)	0.0132 (6)	0.0026 (5)	−0.0013 (5)	0.0019 (5)
C13	0.0162 (8)	0.0151 (8)	0.0122 (7)	0.0002 (6)	−0.0014 (6)	0.0048 (6)
O13	0.0290 (7)	0.0153 (6)	0.0152 (6)	−0.0003 (5)	−0.0051 (5)	−0.0007 (5)
C14	0.0135 (8)	0.0181 (8)	0.0128 (7)	0.0004 (6)	−0.0038 (6)	0.0056 (6)
C15	0.0092 (7)	0.0156 (7)	0.0129 (7)	0.0018 (6)	−0.0003 (6)	0.0081 (6)
C16	0.0111 (7)	0.0178 (8)	0.0173 (8)	0.0052 (6)	0.0008 (6)	0.0077 (7)
N17	0.0093 (6)	0.0100 (6)	0.0116 (6)	0.0015 (5)	−0.0019 (5)	0.0021 (5)
O8	0.0199 (7)	0.0198 (7)	0.0130 (6)	0.0092 (5)	−0.0041 (5)	0.0004 (5)

### Geometric parameters (Å, º)

**Table:**

S1—O5	1.4759 (14)	N7—H7	0.83 (3)
S1—O7	1.4805 (14)	C11—N11	1.318 (2)
S1—O6	1.4843 (14)	C11—N17	1.342 (2)
S1—O4	1.4949 (16)	C11—N12	1.349 (2)
C1—N1	1.315 (2)	N11—H11A	0.87 (3)
C1—N7	1.349 (2)	N11—H11B	0.89 (3)
C1—N2	1.353 (2)	N12—C13	1.402 (2)
N1—H1A	0.87 (3)	N12—H12	0.91 (3)
N1—H1B	0.83 (3)	C13—O13	1.226 (2)
N2—C3	1.403 (2)	C13—C14	1.437 (3)
N2—H2	0.91 (3)	C14—C15	1.351 (2)
C3—O3	1.229 (2)	C14—H14	0.94 (3)
C3—C4	1.443 (2)	C15—N17	1.383 (2)
C4—C5	1.352 (2)	C15—C16	1.492 (3)
C4—H4	0.97 (2)	C16—H16A	0.95 (2)
C5—N7	1.382 (2)	C16—H16B	0.972 (19)
C5—C6	1.491 (2)	C16—H16C	0.953 (19)
C6—H6A	0.98 (2)	N17—H17	0.83 (3)
C6—H6B	0.92 (2)	O8—H8A	0.86 (3)
C6—H6C	0.97 (2)	O8—H8B	0.82 (3)
			
O5—S1—O7	109.31 (8)	C1—N7—H7	120.5 (19)
O5—S1—O6	109.99 (8)	C5—N7—H7	117.9 (19)
O7—S1—O6	110.24 (9)	N11—C11—N17	120.64 (15)
O5—S1—O4	108.97 (8)	N11—C11—N12	120.77 (16)
O7—S1—O4	108.81 (7)	N17—C11—N12	118.59 (15)
O6—S1—O4	109.49 (8)	C11—N11—H11A	119.9 (18)
N1—C1—N7	120.88 (15)	C11—N11—H11B	119.6 (17)
N1—C1—N2	120.63 (15)	H11A—N11—H11B	120 (2)
N7—C1—N2	118.48 (15)	C11—N12—C13	123.73 (15)
C1—N1—H1A	122.7 (16)	C11—N12—H12	117.5 (17)
C1—N1—H1B	117.4 (17)	C13—N12—H12	118.8 (17)
H1A—N1—H1B	120 (2)	O13—C13—N12	118.40 (17)
C1—N2—C3	124.22 (14)	O13—C13—C14	126.54 (17)
C1—N2—H2	116.8 (17)	N12—C13—C14	115.06 (15)
C3—N2—H2	118.0 (17)	C15—C14—C13	120.77 (16)
O3—C3—N2	119.39 (15)	C15—C14—H14	121.2 (15)
O3—C3—C4	125.98 (16)	C13—C14—H14	118.0 (15)
N2—C3—C4	114.61 (14)	C14—C15—N17	119.59 (16)
C5—C4—C3	120.56 (15)	C14—C15—C16	125.40 (16)
C5—C4—H4	120.1 (14)	N17—C15—C16	115.00 (15)
C3—C4—H4	119.2 (14)	C15—C16—H16A	110.6 (13)
C4—C5—N7	120.36 (15)	C15—C16—H16B	109.0 (12)
C4—C5—C6	123.67 (15)	H16A—C16—H16B	111 (2)
N7—C5—C6	115.96 (15)	C15—C16—H16C	110.2 (12)
C5—C6—H6A	108.6 (13)	H16A—C16—H16C	110 (2)
C5—C6—H6B	112.1 (14)	H16B—C16—H16C	106 (2)
H6A—C6—H6B	108 (2)	C11—N17—C15	122.20 (15)
C5—C6—H6C	109.4 (14)	C11—N17—H17	119.0 (19)
H6A—C6—H6C	111 (2)	C15—N17—H17	118.7 (19)
H6B—C6—H6C	108 (2)	H8A—O8—H8B	108 (3)
C1—N7—C5	121.60 (14)		

### Hydrogen-bond geometry (Å, º)

**Table:**

*D*—H···*A*	*D*—H	H···*A*	*D*···*A*	*D*—H···*A*
N1—H1*A*···O6^i^	0.87 (3)	2.02 (3)	2.856 (3)	161 (3)
N1—H1*B*···O4^ii^	0.83 (3)	2.01 (3)	2.847 (4)	178 (2)
N2—H2···O6^iii^	0.91 (3)	1.88 (2)	2.749 (4)	161 (2)
N7—H7···O7^ii^	0.83 (3)	1.91 (3)	2.730 (4)	170 (3)
N11—H11*A*···O5^iii^	0.87 (3)	1.91 (3)	2.782 (6)	178 (3)
N11—H11*B*···O5^iv^	0.89 (3)	2.07 (3)	2.767 (3)	134 (2)
N12—H12···O8^iv^	0.92 (3)	1.87 (3)	2.771 (4)	170 (3)
N17—H17···O4^iii^	0.82 (3)	1.92 (3)	2.746 (6)	177 (3)
O8—H8*A*···O13^v^	0.86 (3)	1.94 (3)	2.750 (5)	158 (3)
O8—H8*B*···O7	0.83 (3)	2.02 (3)	2.839 (4)	173 (3)
C4—H4···O3^vi^	0.97 (2)	2.54 (2)	3.485 (3)	165 (2)
C6—H6*C*···O13^ii^	0.97	2.51	3.440 (3)	162 (2)
C16—H16*A*···O3^vii^	0.95	2.58	3.499 (5)	163 (2)

Symmetry codes: (i) *x*−1, *y*−1, *z*; (ii) *x*, *y*−1, *z*; (iii) −*x*, −*y*+1, −*z*; (iv) *x*−1, *y*, *z*; (v) −*x*+1, −*y*+2, −*z*+1; (vi) −*x*, −*y*+1, −*z*+1; (vii) *x*+1, *y*, *z*.

## References

[bb1] Bernstein, J., Davis, R. E., Shimoni, L. & Chang, N.-L. (1995). *Angew. Chem. Int. Ed. Engl.* **34**, 1555–1573.

[bb2] Bruker (2007). *APEX2* and *SAINT* Bruker AXS Inc., Madison, Wisconsin, USA.

[bb3] Bruker (2012). *SAINT* Bruker AXS Inc., Madison, Wisconsin, USA.

[bb4] Das, B. K., Bora, S. J., Bhattacharyya, M. K. & Barman, R. K. (2009). *Acta Cryst.* B**65**, 467–473. 10.1107/S0108768109021090 19617682

[bb5] Farrugia, L. J. (2012). *J. Appl. Cryst.* **45**, 849–854.

[bb6] Fragoso, P. T., Carneiro, J. W. M. & Vargas, M. D. (2010). *J. Mol. Model.* **16**, 825–829. 10.1007/s00894-009-0579-x 19756783

[bb7] Grobelny, R., Glowiak, T., Mrozinski, J., Baran, W. & Tomasik, P. (1995). *Pol. J. Chem.* **69**, 559–565.

[bb8] Molloy, K. C. (1988). *Inorg. Chem. Acta*, **141**, 151–333.

[bb9] Norquist, A. J., Doran, M. B. & O’Hare, D. (2005). *Acta Cryst.* E**61**, m807–m810.

[bb10] Sheldrick, G. M. (2008). *Acta Cryst.* A**64**, 112–122. 10.1107/S0108767307043930 18156677

[bb11] Vollano, J. F., Day, R. O., Rau, D. N., Chadrasekhar, V. & Holmes, R. R. (1984). *Inorg. Chem.* **23**, 3152–3155.

[bb12] Yang, R. N., Wang, D. M., Hou, Y. M., Xue, B. Y., Jin, D. M., chen, L. R. & Luo, B. S. (1995). *Acta Chem. Scand.* **49**, 771–773.

